# Thyroid cancer among female workers in Korea, 2007–2015

**DOI:** 10.1186/s40557-018-0259-3

**Published:** 2018-07-16

**Authors:** Seonghoon Kang, Jinho Song, Taehwan Koh, One Park, Jong-Tae Park, Won-Jin Lee

**Affiliations:** 1 Department of Occupational and Environmental Medicine, Ansan Hospital, Korea University Medical Center, Korea University, Gyeonggi-do, South Korea; 2 Graduate School of Public Health, Korea University, Seongbuk-gu, Seoul, South Korea; 3 Department of Preventive Medicine, Korea University College of Medicine, 73, Inchon-ro, Seongbuk-gu, Seoul, 02855 South Korea

**Keywords:** Women, Working, Thyroid Neoplasms, Industry

## Abstract

**Background:**

Prevalence of thyroid cancer has been increasing rapidly worldwide, especially among women. There has been a debate as to whether such an increase represents consequences of over-diagnosis or a true increase. To find the occupational risk of Korean female workers in different industry sectors, we analyzed the data of Korean female workers.

**Methods:**

National Female Worker Cohort data that contain information on total female workers were used for our analysis of prevalence of thyroid cancer (C73 according to KCD-5, 6 code) derived from National Health Insurance data. By combining industrial codes from National Health Insurance Service and those from Korea Workers’ Compensation and Welfare Service, the classification of industrial codes became to consist of the total of thirty three representing both non-office (NO) and office (O) categories. Both an internal comparison among female workers within the cohort and an external comparison to compare female workers with Korean general female population were carried out.

**Results:**

Among 149,258 female workers, 2,641 cases of thyroid cancers were identified. Differences in prevalence of thyroid cancer between female workers (40.5%) and general Korean female population (32.6%) were observed; however, the differences in prevalence of thyroid cancer between NO workers and O workers were not apparent. An analysis involving workers in Financial and insurance activities sector revealed that, standardized prevalence rate (SPR) of both NO (2.96, 95% CI = 2.01–4.20) and O workers (3.68, 95% CI = 3.10–4.33) increased significantly and that an AOR (adjusted odds ratio) increased marginally (1.38, 95% CI = 0.97–1.96). Further, when stratified in respect to the duration of employment, an AOR of female workers having been employed for more than 8 years showed a significant increase (1.63, 95% CI = 1.07–2.49).

**Conclusions:**

Female workers had a higher risk of thyroid cancer than general female population but the difference between NO workers and O workers was not found to be significant in most industrial sectors. Further studies using data with information regarding specific occupational exposures are needed.

**Electronic supplementary material:**

The online version of this article (10.1186/s40557-018-0259-3) contains supplementary material, which is available to authorized users.

## Background

Thyroid cancer has been increasing rapidly worldwide, especially among women [1]. According to GLOBOCAN 2012, the estimated incidence of thyroid cancer was 229,923 persons, accounting for 3.5% of cancer among women and thyroid cancer presented as the seventh most common cancer for women worldwide (Female: Male sex ratio 3). In North America, the age-standardized incidence rate of thyroid cancer was estimated to 20.0 per 100,000 for female [2]. In Korea, for the past decade, the incidence of thyroid cancer increased 1.25 times each year [3]; therefore, thyroid cancer set itself as the most common cancer among women, accounting for 19.4% of total cancers, followed by breast, colorectal, stomach, lung cancer in 2015. The age-standardized incidence was 66.3 per 100,000 for women (Female: Male sex ratio 4) [4].

The cause of the global increase is still debated. Some experts believe that the sharp increase occurred due to the recent development of sensitive diagnostic procedures, as it was the case with prostate cancer in Northern America and Western European countries [5]. Especially in Korea, ‘over-diagnosis’ is believed to be one of the possible explanation of the increase [6], contributing to almost a quarter of the increase [7]. However, others argue that such increase has occurred due to an increased use of radiation in the field of medicine, thyroid-specific environmental carcinogens that may be unrecognized as of now and lifestyle changes in the setting of growing urbanization [1, 8]. The known risk factors of thyroid cancer are female gender [9], an exposure of ionizing radiation during childhood, an increased dietary intake of iodine, obesity, family history of thyroid cancer and medical history of benign thyroid disease [10–13].

Enough attention needs to be paid to occupational cancer of women as much as to that of men [14]. Industrialization and globalization are two strong forces that have changed patterns of women employment worldwide [15]. According to National Statistical Office of Korea (KNSO), in the year of 2017, the employed workforce is now 50.2% women, and the primary source of support of the household is 30.3% women in 2016 [16]. However, many studies on occupational risk have been conducted only on male workforce, based on an erroneous belief that work undertaken by female workforce tend to be safer. The percentage of studies on occupational cancer has increased from 39 to 62% between 1999 and 2009; however, only 10% of those focused on women [14]. Substantial gender-based differences in occupational factors such as different tasks [17, 18] and biological susceptibilities [19, 20] and difference in non-occupational factors such as the use of tobacco and alcohol consumption [21] reshape the risk of occupational cancer of women in respect to that of men.

Although women workforce and its occupational exposures have increased rapidly, thyroid cancer, the most common cancer among women has not been given a focus as occupational cancer in Korea. Hence, studies regarding thyroid cancer among female workers are warranted.

The aim of our study is to find the occupational risk of thyroid cancer among female workers of various industrial sectors by estimating the frequency of thyroid cancer for Non-office (NO) workers and comparing it to that of office (O) workers as well as that of general women population.

## Methods

### Study population

National Female Worker Cohort data used in our study is the data collected from the year of 2007; they are on 185,144 female workers who accounted for 5% of 3,710,000 female workers (who, at the end of December, 2007, fall between the age of 15 and 64 and at the same time are categorized as “the workplace-insured” by the standard set by National health insurance). These workers were then followed up to the year of 2015, until which no influx of study population occurred; accounting for losses of eligibility due to death, the size of the cohort decreased to 179,420 female workers by the year of 2015. For our study, disease codes for which disability benefit was claimed, information regarding categorization of study objects based on a) NO/O workers classification, b) main industry codes, c) income decile and health data from General Health Examination and Life Turning Point Health Examination were used.

### Cancer definition

For our study, patients diagnosed with thyroid cancer were defined as those patients whose main diagnosis on their medical bill/statement was designated as C73: Malignant neoplasm of thyroid gland in accordance with Korean Standard Classification of Diseases (KCD-5,6). This meant that both patients who were diagnosed with thyroid cancer for the first time during the year of 2007 and 2015 and patients who have been diagnosed with thyroid cancer before 2007 and accessed medical services during the same period for a routine check-up are included in our count. Patients who were diagnosed with multiple primary cancers or secondary cancer were excluded. No information regarding as to when the actual diagnosis of cancer has been made is not included in the original set of data.

### Exposure assessment

As for the classification of industries, the classification that National Health Insurance Service has produced and expanded on by combining industrial codes from National Health Insurance Service and those from Korea Workers’ Compensation and Welfare Service was used. Among 21 industrial divisions provided by 10th Korea Standard Industry Code (KSIC), division A (Agriculture, forestry and fishing) and division B (Mining and quarrying) were bundled together, and division D (Electricity, gas, steam and air conditioning supply) and division E (Water supply: sewage, waste management, materials recovery) were bundled together to form 19 modified industrial divisions. Then, division C (Manufacturing) among those 19 modified industrial divisions was subdivided into 15 industrial sectors, resulting in 33 industrial sectors in whole. In addition, a job categorical code (the one that divides NO/O workers) were also utilized. Since the results of General Health Examination and Life Turning Point Health Examination reveal whether a worker is categorized as a NO/O worker, those results of exams in the year of 2007 or the year that is the closest to 2007 were used as references to determine a job categorical code for each worker. As for continuous employment, if workers’ cohort data revealed the same and repeating values of industrial sector codes from 2007, the workers are considered to have done “continuous employment.” Information regarding industries or the duration of employment prior to 2007 was unavailable; hence the data between 2007 and 2015 were solely used.

### Statistical analyses

χ2 tests were used to compare the distribution of risk factors among female workers with thyroid cancer to those without thyroid cancer. χ2 tests were carried out on three groups: a group of total female workers as well as two subgroups of NO and O female workers. Among 33 industrial codes from each of NO and O category, industrial codes with more than 5 counts of workers diagnosed with thyroid cancer were searched and selected for further analysis.

To eliminate effects of age structure of Cohort, Standardized Prevalence Ratio (SPR) was calculated by indirect method for each of 33 industrial sectors. Period prevalence rate of female workers in our cohort and reference were calculated using an age bracket of 5 years (15–19, 20–24, …, 60–64). Reference prevalence rate was calculated using the numerator as the number of patients newly diagnosed with thyroid cancer between 1999 and 2014 as evinced from the Annual Report of the Korea Central Cancer Registry and the denominator as the number of general women population at 2010 published by the KNSO [22]. Although the reference prevalence rate was calculated using the date from the year of 1999 and 2014, the cohort data between the year of 2007 and 2014 were only used regarding thyroid cancer cases. The classification table was calculated by Poisson Regression analysis of cancer prevalence [23]. To see the difference in the SPR between NO and O workers, Standardized Prevalence Rate Ratio (SRR) was calculated using office workers as the comparison group.

Multiple logistic analyses were conducted, controlling for confounding risk factors, to calculate Adjusted Odds Ratio (AOR) of thyroid cancer (and their 95% Confidence interval) by comparing the prevalence of thyroid cancer of NO workers from each of 33 industrial sectors and that of O workers in whole. Based on prior knowledge from pre-existing literatures and statistical analyses, Odds Ratio (OR) was adjusted for age (continuous), smoking (Non-, ex-, current), alcohol consumption (0, 1–2, ≥3 times/week), income decile (0, 1, …, 10), Body Mass Index (BMI) (< 18.5, 18.5–24.9, 25.0–29.9, ≥30) and physical activity (never, ≤2, ≥3 times/week); the data gathered in the year of 2007 or the year closest to 2007 were used for analysis. Much of the data gathering for many risk factors were collected during General Health Examination and Life Turning Point Health Examination. BMI was calculated by weight in kilograms divided by the square of height in meters. Content of self-reported questionnaire of General Health examination regarding level of physical activity slightly changed in the year of 2009. As such, regarding frequency of physical activity, the variable of “the number of physical activities of moderate intensity that last more than 30 minutes per week” (from the year of 2009) was deemed equivalent to that of “the number of physical activities per week” (before the year of 2009). Since all of the variables were significant in the univariable analysis, they were included in the final analysis. Further, a stratified analysis by duration of employment (≤3, 3–8, > 8 years) was conducted as well.

To examine the dose-response trend, and AOR for different duration of exposures (≤3, 3–8, > 8 years) were calculated by comparing the prevalence of thyroid cancer of NO workers and that of O workers regarding each respective duration of work, from which p-trend was derived. Further, a stratified analysis by size of enterprises in terms of number of workers involved (< 100, ≥100 workers) was conducted as well.

Statistical analyses were performed with using SAS Enterprise Guide Version 7.1., and the α-level for significance tests was set at 0.05.

## Results

Total of 149,258, of which 71,176/78,082 were classified as NO/O respectively, were enrolled, after excluding those workers whose the data between the year of 2007 and 2015 regarding their occupation, job categories (NO/O), confounders were missing (Table 1). Among 149,258 workers who were enrolled, 2614 workers, of which 1177/1437 workers were classified as NO/O workers respectively, were diagnosed with thyroid cancer. The total number of patients diagnosed with any form of cancer was 6451, and the number of patients diagnosed with thyroid cancer amounted to 40.5% of the total. Workers diagnosed with thyroid cancer (of both NO and O categories) tend to be involved in more physical activities than workers without thyroid cancer. Workers diagnosed with thyroid cancer had tendencies to be more obese, non-smoking, and non-alcoholic. As far as income structure of total workers is concerned, NO workers of lower income deciles and O workers of higher income deciles had more tendency to be diagnosed with thyroid cancer. As far as the structure of industry is concerned, top three industries with most NO workers were Human health and social work activities (15.4%)/Education (10.3%)/Wholesale and retail trade (9.0%). The frequency of thyroid cancer among NO workers followed the same order as well. Top three industries with most O workers were Public administration and defense; compulsory social security (23.8%)/Education (10.4%)/Wholesale and retail (9.9%). However, the frequency of thyroid cancer among O workers followed a decreasing order of Public administration and defense; compulsory social security (29.0%)/Financial and insurance activities (10.6%)/Education (10.2%).

**Table 1:** Distribution of risk factors for thyroid cancer among non-office, and office) workers

	Total			Non-office			Office		
	All (%)	Cancer (%)	χ2 test^a^	All	Cancer	χ2 test^a^	All	Cancer	χ2 test^a^
	*n* = 149,258	*n* = 2614 (1.8)		*n* = 71,176	*n* = 1177 (1.7)		*n* = 78,082	*n* = 1437 (1.8)	
Age(years)			*p* = < 0.001			*p* = < 0.001			*p* = < 0.001
15–19	990 (0.7)	3 (0.1)		622 (0.9)	2 (0.2)		368 (0.5)	1 (0.1)	
20–24	17,402 (11.7)	121 (4.6)		8402 (11.8)	53 (4.5)		9000 (11.5)	68 (4.7)	
25–29	33,063 (22.2)	413 (15.8)		13,758 (19.3)	172 (14.6)		19,305 (24.7)	241 (16.8)	
30–34	23,160 (15.5)	376 (14.4)		8734 (12.3)	131 (11.1)		14,426 (18.5)	245 (17.0)	
35–39	21,673 (14.5)	448 (17.1)		8271 (11.6)	154 (13.1)		13,402 (17.2)	294 (20.5)	
40–44	18,364 (12.3)	421 (16.1)		9670 (13.6)	195 (16.6)		8694 (11.1)	226 (15.7)	
45–49	16,458 (11.0)	422 (16.1)		9979 (14.0)	230 (19.5)		6479 (8.3)	192 (13.4)	
50–54	10,173 (6.8)	247 (9.4)		6561 (9.2)	134 (11.4)		3612 (4.6)	113 (7.9)	
55–59	5446 (3.6)	114 (4.4)		3462 (4.9)	69 (5.9)		1984 (2.5)	45 (3.1)	
60–64	2529 (1.7)	49 (1.9)		1717 (2.4)	37 (3.1)		812 (1.0)	12 (0.8)	
Income decile			*p* = < 0.001			*p* = 0.0157			*p* = < 0.001
0 (lowest)	0 (0.0)	0 (0.0)		0 (0.0)	0 (0.0)		0 (0.0)	0 (0.0)	
1	26,560 (17.8)	486 (18.6)		13,676 (19.2)	228 (19.4)		12,884 (16.5)	258 (18.0)	
2	22,208 (14.9)	358 (13.7)		12,365 (17.4)	206 (17.5)		9843 (12.6)	152 (10.6)	
3	19,081 (12.8)	289 (11.1)		10,877 (15.3)	158 (13.4)		8204 (10.5)	131 (9.1)	
4	19,096 (12.8)	246 (9.4)		9956 (14.0)	144 (12.2)		9140 (11.7)	102 (7.1)	
5	15,773 (10.6)	256 (9.8)		8001 (11.2)	130 (11.0)		7772 (10.0)	126 (8.8)	
6	13,051 (8.7)	219 (8.4)		5841 (8.2)	92 (7.8)		7210 (9.2)	127 (8.8)	
7	12,740 (8.5)	240 (9.2)		4729 (6.6)	95 (8.1)		8011 (10.3)	145 (10.1)	
8	11,420 (7.7)	248 (9.5)		3601 (5.1)	75 (6.4)		7819 (10.0)	173 (12.0)	
9	9329 (6.3)	272 (10.4)		2130 (3.0)	49 (4.2)		7199 (9.2)	223 (15.5)	
10 (highest)	0 (0.0)	0 (0.0)		0 (0.0)	0 (0.0)		0 (0.0)	0 (0.0)	
Smoking			*p* = < 0.001			*p* = 0.0075			*p* = 0.0011
Never	142,373 (95.4)	2539 (97.1)		67,782 (95.2)	1143 (97.1)		74,591 (95.5)	1396 (97.1)	
Former	2360 (1.6)	32 (1.2)		1031 (1.4)	8 (0.7)		1329 (1.7)	24 (1.7)	
Current	4525 (3.0)	43 (1.6)		2363 (3.3)	26 (2.2)		2162 (2.8)	17 (1.2)	
Alcohol(times/week)			*p* = < 0.001			*p* = 0.0072			*p* = < 0.001
0	120,686 (80.9)	2211 (84.6)		58,419 (82.1)	1007 (85.6)		62,267 (79.7)	1204 (83.8)	
1–2	24,952 (16.7)	366 (14.0)		11,005 (15.5)	147 (12.5)		13,947 (17.9)	219 (15.2)	
≥ 3	3620 (2.4)	37 (1.4)		1752 (2.5)	23 (2.0)		1868 (2.4)	14 (1.0)	
BMI((kg/m^2^)			*p* = < 0.001			*p* = < 0.001			*p* = < 0.001
< 18.5	15,365 (10.3)	170 (6.5)		6563 (9.2)	61 (5.2)		8802 (11.3)	109 (7.6)	
18.5–24.9	110,945 (74.3)	1936 (74.1)		52,122 (73.2)	858 (72.9)		58,823 (75.3)	1078 (75.0)	
25.0–29.9	19,933 (13.4)	447 (17.1)		10,922 (15.3)	238 (20.2)		9011 (11.5)	209 (14.5)	
≥ 30	3015 (2.0)	61 (2.3)		1569 (2.2)	20 (1.7)		1446 (1.9)	41 (2.9)	
Physical activity			*p* = < 0.001			*p* = 0.0818			*p* = < 0.001
never	95,449 (63.9)	1556 (59.5)		46,349 (65.1)	738 (62.7)		49,100 (62.9)	818 (56.9)	
1~ 2 times/week	35,381 (23.7)	678 (25.9)		16,246 (22.8)	274 (23.3)		19,135 (24.5)	404 (28.1)	
≥ 3 times/week	18,428 (12.3)	380 (14.5)		8581 (12.1)	165 (14.0)		9847 (12.6)	215 (15.0)	
Industry sectors			*p* = < 0.001			*p* = 0.6728			*p* = < 0.001
Agriculture, forestry, fishing, mining and quarrying	492 (0.3)	13 (0.5)		297 (0.4)	7 (0.6)		195 (0.2)	6 (0.4)	
Manufacture of beverages and food products	2401 (1.6)	43 (1.6)		1790 (2.5)	36 (3.1)		611 (0.8)	7 (0.5)	
Manufacture of textiles and apparel	2985 (2.0)	52 (2.0)		1836 (2.6)	30 (2.5)		1149 (1.5)	22 (1.5)	
Printing and printing related industry	1011 (0.7)	12 (0.5)		367 (0.5)	4 (0.3)		644 (0.8)	8 (0.6)	
Manufacture of coke, briquettes, refined petroleum, chemicals and chemical products	1174 (0.8)	22 (0.8)		733 (1.0)	10 (0.8)		441 (0.6)	12 (0.8)	
Manufacture of pharmaceuticals, medicinal chemical and botanical products	144 (0.1)	2 (0.1)		97 (0.1)	2 (0.2)		47 (0.1)	0 (0.0)	
Manufacture of rubber and plastics products	885 (0.6)	12 (0.5)		605 (0.9)	7 (0.6)		280 (0.4)	5 (0.3)	
Manufacture of glass and non-metallic mineral products	332 (0.2)	4 (0.2)		193 (0.3)	1 (0.1)		139 (0.2)	3 (0.2)	
Manufacture of basic metals	1537 (1.0)	22 (0.8)		940 (1.3)	16 (1.4)		597 (0.8)	6 (0.4)	
Manufacture of electronic components, computer; visual, sounding and communication equipment	4605 (3.1)	60 (2.3)		3602 (5.1)	50 (4.2)		1003 (1.3)	10 (0.7)	
Manufacture of medical, precision and optical instruments, watches and clocks	314 (0.2)	3 (0.1)		199 (0.3)	0 (0.0)		115 (0.1)	3 (0.2)	
Manufacture of electrical equipment	883 (0.6)	15 (0.6)		549 (0.8)	8 (0.7)		334 (0.4)	7 (0.5)	
Manufacture of machinery and equipment	8727 (5.8)	133 (5.1)		6067 (8.5)	103 (8.8)		2660 (3.4)	30 (2.1)	
Manufacture of motor vehicles, trailers and semitrailers, and transport equipment	2433 (1.6)	37 (1.4)		1820 (2.6)	29 (2.5)		613 (0.8)	8 (0.6)	
Manufacture of wood, products of wood, cork and furniture	720 (0.5)	16 (0.6)		421 (0.6)	7 (0.6)		299 (0.4)	9 (0.6)	
Other manufacturing	4244 (2.8)	57 (2.2)		3101 (4.4)	39 (3.3)		1143 (1.5)	18 (1.3)	
Electricity, gas, steam, sewage, wastewater treatment services	602 (0.4)	11 (0.4)		237 (0.3)	2 (0.2)		365 (0.5)	9 (0.6)	
Construction	4069 (2.7)	63 (2.4)		1207 (1.7)	19 (1.6)		2862 (3.7)	44 (3.1)	
Wholesale and retail trade	14,168 (9.5)	262 (10.0)		6436 (9.0)	122 (10.4)		7732 (9.9)	140 (9.7)	
Transportation	2720 (1.8)	42 (1.6)		1271 (1.8)	21 (1.8)		1449 (1.9)	21 (1.5)	
Accommodation and food service activities	4038 (2.7)	68 (2.6)		2475 (3.5)	44 (3.7)		1563 (2.0)	24 (1.7)	
Publishing activities, motion picture, broadcasting activities, telecommunications, information service activities	3527 (2.4)	58 (2.2)		1090 (1.5)	19 (1.6)		2437 (3.1)	39 (2.7)	
Financial and insurance activities	7260 (4.9)	186 (7.1)		1450 (2.0)	33 (2.8)		5810 (7.4)	153 (10.6)	
Real estate activities and rental and leasing activities	5168 (3.5)	91 (3.5)		2558 (3.6)	47 (4.0)		2610 (3.3)	44 (3.1)	
Professional, scientific and technical activities	5022 (3.4)	70 (2.7)		1383 (1.9)	20 (1.7)		3639 (4.7)	50 (3.5)	
Business facilities management and business support services	7722 (5.2)	111 (4.2)		4686 (6.6)	66 (5.6)		3036 (3.9)	45 (3.1)	
Public administration and defense; compulsory social security	21,400 (14.3)	476 (18.2)		2805 (3.9)	59 (5.0)		18,595 (23.8)	417 (29.0)	
Education	15,447 (10.3)	273 (10.4)		7329 (10.3)	126 (10.7)		8118 (10.4)	147 (10.2)	
Human health and social work activities	16,158 (10.8)	251 (9.6)		10,948 (15.4)	174 (14.8)		5210 (6.7)	77 (5.4)	
Arts, sports and recreation related services	1962 (1.3)	31 (1.2)		1066 (1.5)	17 (1.4)		896 (1.1)	14 (1.0)	
Membership organizations, repair and other personal services	6357 (4.3)	107 (4.1)		3238 (4.5)	53 (4.5)		3119 (4.0)	54 (3.8)	
Activities of households as employers	575 (0.4)	8 (0.3)		335 (0.5)	5 (0.4)		240 (0.3)	3 (0.2)	
Activities of extraterritorial organizations and bodies	176 (0.1)	3 (0.1)		45 (0.1)	1 (0.1)		131 (0.2)	2 (0.1)	

^a^χ2 test comparing the distribution in all sample and thyroid cancer cases

The difference in prevalence of thyroid cancer between NO workers and O workers was not apparent (Table 2). Among NO workers, those from Financial and insurance activities/Publishing activities, motion picture, broadcasting activities, telecommunications, information service activities/Human health and social work activities exhibited a higher SPR. Among O workers, those from Financial and insurance activities/Agriculture, forestry, fishing, mining and quarrying/Manufacture of coke, briquettes, refined petroleum, chemicals and chemical products exhibited a higher SPR. Workers from four industrial sectors: Accommodation and food service activities/Manufacture of electronic components, computer; visual, sounding and communication equipment/Manufacture of basic metals/Manufacture of beverages and food products exhibited an increased yet statistically insignificant SRR. Workers from five industrial sectors such as Manufacture of textiles and apparel/Wholesale and retail/Business facilities management and business support service/Public administration and defense; compulsory social security/Membership organizations, repair and other personal services exhibited a decreased SRR at a statistically significant level. We further analyzed Standardized Incidence Ratio (SIR) excluding the cases in 2007 which is considered to prevalence, however, the result was similar to the SPR (See Supplementary Table 1, Additional File 1).

**Table 2:** Standardized prevalence ratio and standardized rate ratio by industrial sectors and job categories (Reference: Korean female general population)

	Total				Non-office				Office				Non-office/Office		
Industry sectors	Cases	SPR	95% CI		Cases	SPR	95% CI		Cases	SPR	95% CI		SRR	95% CI	
Agriculture, forestry, fishing, mining and quarrying	13	1.90	1.01	3.25	7	1.48	0.59	3.05	6	2.86	1.05	6.22	0.52	0.17	1.54
Manufacture of beverages and food products	43	1.28	0.92	1.72	34	1.30	0.90	1.82	9	1.20	0.55	2.27	1.08	0.52	2.26
Manufacture of textiles and apparel	48	1.21	0.90	1.61	26	0.96	0.63	1.41	22	1.76	1.10	2.66	0.55	0.31	0.97
Manufacture of coke, briquettes, refined petroleum, chemicals and chemical products	20	1.94	1.18	3.00	9	1.42	0.65	2.70	11	2.75	1.38	4.93	0.52	0.21	1.25
Manufacture of rubber and plastics products	12	0.99	0.51	1.73	7	0.79	0.32	1.64	5	1.50	0.49	3.50	0.53	0.17	1.67
Manufacture of basic metals	23	1.15	0.73	1.73	16	1.20	0.68	1.94	7	1.06	0.43	2.19	1.13	0.46	2.73
Manufacture of electronic components, computer; visual, sounding and communication equipment	53	1.35	1.01	1.77	43	1.39	1.01	1.88	10	1.19	0.57	2.18	1.18	0.59	2.34
Manufacture of electrical equipment	13	1.28	0.68	2.20	6	0.85	0.31	1.84	7	2.31	0.93	4.75	0.37	0.12	1.09
Manufacture of machinery and equipment	127	1.24	1.03	1.47	96	1.20	0.97	1.47	31	1.35	0.92	1.92	0.89	0.59	1.33
Manufacture of motor vehicles, trailers and semitrailers, and transport equipment	38	1.20	0.85	1.65	30	1.16	0.78	1.66	8	1.37	0.59	2.70	0.85	0.39	1.85
Manufacture of wood, products of wood, cork and furniture	16	1.55	0.88	2.51	7	1.03	0.41	2.13	9	2.53	1.16	4.81	0.41	0.15	1.09
Other manufacturing	52	1.57	1.17	2.06	33	1.45	1.00	2.03	19	1.84	1.11	2.88	0.79	0.45	1.38
Construction	68	1.54	1.19	1.95	18	1.12	0.66	1.76	50	1.78	1.32	2.34	0.63	0.37	1.08
Wholesale and retail trade	262	1.76	1.55	1.99	116	1.54	1.27	1.84	146	1.99	1.68	2.34	0.77	0.60	0.98
Transportation	42	1.74	1.25	2.35	21	1.69	1.05	2.58	21	1.79	1.11	2.74	0.94	0.52	1.73
Accommodation and food service activities	67	1.12	0.87	1.42	45	1.24	0.90	1.66	22	0.94	0.59	1.43	1.31	0.79	2.19
Publishing activities, motion picture, broadcasting activities, telecommunications, information service activities	57	2.32	1.76	3.01	18	2.06	1.22	3.26	39	2.46	1.75	3.37	0.84	0.48	1.46
Financial and insurance activities	173	3.52	3.02	4.09	31	2.96	2.01	4.20	142	3.68	3.10	4.33	0.80	0.55	1.19
Real estate activities and rental and leasing activities	87	1.17	0.93	1.44	46	1.01	0.74	1.35	41	1.40	1.01	1.90	0.72	0.47	1.10
Professional, scientific and technical activities	70	1.79	1.39	2.26	18	1.30	0.77	2.05	52	2.06	1.54	2.70	0.63	0.37	1.08
Business facilities management and business support services	105	1.13	0.92	1.37	65	0.99	0.76	1.26	40	1.48	1.06	2.01	0.67	0.45	0.99
Public administration and defense; compulsory social security	456	1.86	1.69	2.04	55	1.39	1.04	1.80	401	1.95	1.77	2.15	0.71	0.53	0.94
Education	277	1.74	1.54	1.96	127	1.61	1.34	1.91	150	1.87	1.59	2.20	0.86	0.68	1.09
Human health and social work activities	240	1.72	1.51	1.96	164	1.71	1.46	2.00	76	1.74	1.37	2.18	0.98	0.75	1.29
Arts, sports and recreation related services	29	1.31	0.88	1.88	16	1.10	0.63	1.79	13	1.70	0.90	2.90	0.65	0.31	1.35
Membership organizations, repair and other personal services	104	1.35	1.10	1.64	50	1.09	0.81	1.44	54	1.74	1.31	2.27	0.63	0.43	0.92

For age-standardized external comparison, the total of 155,842 workers, of which 73,226/82,616 workers were classified as non-office/office respectively, were enrolled, after excluding those workers who either have deceased or for whom the data between the year of 2007 and 2014 regarding their occupation and information on categorization of occupation (non-office/office) did not exist. Among them, 2537 workers, of which 1120/1417 workers were classified as non-office/office respectively, were diagnosed with thyroid cancer

An AOR of Business facilities management and business support services sector was decreased at a statistically significant level; however, an AOR of Financial and insurance activities was marginally increased (1.38, 95% CI = 0.97–1.96) and was significantly increased among workers who worked longer than 8 years (1.63, 95% CI = 1.07–2.49) (Table 3).

**Table 3:** Adjusted odd ratio and duration of employment by industrial sectors and job categories (Reference: office workers regarding each respective duration of work)

	Odds Ratio				≤3 year				3–8 year				> 8 year			
Industry sectors	Cases	AOR^a^	95% CI		Cases	AOR^a^	95% CI		Cases	AOR^a^	95% CI		Cases	AOR^a^	95% CI	
Agriculture, forestry, fishing, mining and quarrying	7	1.11	0.52	2.36	1	0.51	0.07	3.65	3	1.49	0.47	4.75	3	1.33	0.42	4.21
Manufacture of beverages and food products	36	0.97	0.69	1.36	11	1.07	0.58	1.98	9	0.70	0.36	1.38	16	1.14	0.69	1.90
Manufacture of textiles and apparel	30	0.80	0.55	1.16	11	0.97	0.53	1.79	15	1.06	0.63	1.79	4	0.33	0.12	0.90
Manufacture of coke, briquettes, refined petroleum, chemicals and chemical products	10	0.78	0.42	1.45	2	0.55	0.14	2.21	4	1.03	0.38	2.78	4	0.77	0.29	2.08
Manufacture of rubber and plastics products	7	0.56	0.27	1.18	2	0.42	0.10	1.69	2	0.46	0.12	1.88	3	0.90	0.28	2.83
Manufacture of basic metals	16	0.82	0.50	1.35	5	0.67	0.28	1.64	7	1.12	0.53	2.40	4	0.69	0.26	1.86
Manufacture of electronic components, computer; visual, sounding and communication equipment	50	0.83	0.63	1.11	17	0.83	0.51	1.36	15	0.73	0.44	1.24	18	0.97	0.60	1.55
Manufacture of electrical equipment	8	0.74	0.37	1.49	3	0.69	0.22	2.17	4	1.11	0.41	3.02	1	0.37	0.05	2.65
Manufacture of machinery and equipment	103	0.84	0.69	1.03	27	0.69	0.47	1.03	43	0.97	0.70	1.33	33	0.86	0.60	1.23
Manufacture of motor vehicles, trailers and semitrailers, and transport equipment	29	0.75	0.52	1.09	7	0.66	0.31	1.41	6	0.47	0.21	1.05	16	1.04	0.63	1.73
Manufacture of wood, products of wood, cork and furniture	7	0.77	0.36	1.62	2	0.66	0.16	2.69	2	0.67	0.16	2.71	3	0.97	0.31	3.07
Other manufacturing	39	0.75	0.55	1.04	6	0.45	0.20	1.02	15	0.88	0.52	1.48	18	0.84	0.52	1.35
Construction	19	0.83	0.52	1.31	10	1.10	0.58	2.08	6	0.77	0.34	1.74	3	0.49	0.16	1.52
Wholesale and retail trade	122	1.05	0.87	1.26	36	1.06	0.75	1.51	38	1.00	0.71	1.40	48	1.08	0.80	1.46
Transportation	21	0.87	0.56	1.35	3	0.56	0.18	1.75	4	0.65	0.24	1.76	14	1.12	0.66	1.92
Accommodation and food service activities	44	0.87	0.64	1.19	10	0.64	0.34	1.21	20	0.97	0.61	1.54	14	1.04	0.60	1.78
Publishing activities, motion picture, broadcasting activities, telecommunications, information service activities	19	1.05	0.67	1.66	6	1.03	0.46	2.32	3	0.47	0.15	1.48	10	1.80	0.95	3.41
Financial and insurance activities	33	1.38	0.97	1.96	5	0.98	0.40	2.40	5	0.97	0.40	2.37	23	1.63	1.07	2.49
Real estate activities and rental and leasing activities	47	0.80	0.60	1.08	23	0.92	0.60	1.43	21	0.99	0.63	1.55	3	0.26	0.08	0.82
Professional, scientific and technical activities	20	0.81	0.52	1.26	10	1.21	0.64	2.29	4	0.39	0.14	1.04	6	1.02	0.45	2.30
Business facilities management and business support services	66	0.71	0.55	0.91	28	0.70	0.48	1.04	27	0.75	0.50	1.11	11	0.71	0.39	1.30
Public administration and defense; compulsory social security	59	0.91	0.69	1.18	10	0.73	0.39	1.38	17	0.86	0.52	1.40	32	1.04	0.72	1.48
Education	126	0.93	0.78	1.12	18	0.87	0.54	1.40	61	0.98	0.74	1.28	47	0.87	0.64	1.19
Human health and social work activities	174	0.94	0.80	1.11	35	1.24	0.87	1.77	64	1.01	0.77	1.32	75	0.79	0.62	1.01
Arts, sports and recreation related services	17	0.81	0.50	1.31	8	0.83	0.41	1.69	8	1.06	0.52	2.16	1	0.27	0.04	1.93
Membership organizations, repair and other personal services	53	0.81	0.61	1.07	24	0.84	0.55	1.29	21	0.89	0.57	1.40	8	0.61	0.30	1.24

^a^AOR adjusted for age, smoking, alcohol, BMI, income decile, physical activity

An analysis to discover possible dose-response relationship in respect to the duration of employment was carried out on both NO and O workers from Financial and insurance activities sector; a marginal significance was ascertained (p trend 0.076) (See Supplementary Table 2, Additional File 2). Further, post-stratification analysis by the number of employees also revealed that workers employed in the enterprises of smaller sizes (< 100 workers) faced 1.83 times greater prevalence than those employed in enterprises of bigger sizes (≥100 workers) (See Supplementary Table 3, Additional File 3).

## Discussion

### The difference in thyroid cancer between female workers and general female population

Period prevalence of thyroid cancer was far higher in female workers than in general population. The proportion of thyroid cancer among total kinds of cancer was over 40.5% in our study, although our study involved only a fraction (from age 15 to 73), albeit sizable, of general population. In general female population, the number approximates to 32.6% [4]. The period prevalence of all cancer in our study was 4.3%, while that in general female population was 3.5%. As the proportion of thyroid cancer in respect to total cancer is substantial, an increase in the prevalence of total cancer among female workers owes largely to that of thyroid cancer among them. Possible reasons behind this could be due to health check-up packages including ultrasonography examination (‘over-screening’). An ultrasonography examination on thyroid and liver can be added to health check-up packages with only small extra charges to patients. Plus, since ultrasonography allows a quick and accurate diagnosis, and has thus proven to be cost-effective, medical staffs also suggest it to patients receiving a health check-up. As a result, the proportion of tumors of a small size among all tumors has rapidly increased from 6.1% in 1962 to 43.1% in 2009 while that of tumors of a large size decreased, just as the mortality rate and the recurrence rate have decreased [24]. In 2011, a systemic review revealed that an average prevalence of occult papillary thyroid carcinoma from 7897 autopsies examination turned out to be 7.6% [25]. Frequent exposures to medical radiation from the early age also could be the reason for the increased. Thyroid is very sensitive to radiation, and young age and female gender were found to be risk factors associated with a higher prevalence of papillary thyroid cancer [26]. In Korea, as mentioned earlier, workers have an access to regular health check-ups (that often involve a chest X-ray) from a relatively early age after employment and some of those workers choose to pay extra to take a CT scan or offered as a part of employee benefit program to detect subclinical diseases. On the contrary, females who do not have an occupation (i.e. housewives) or depend on other family members as primary sources of income are offered General Health Examination from the age of 40. Furthermore, the rate of receiving General Health Examination is higher in female workers (85.3%) than in general female population (69.4%), according to Korean statistics in 2015 [27].

### The difference in the prevalence of thyroid cancer between NO workers and O workers

The difference in the prevalence of thyroid cancer between NO workers and O workers was not significant in most industrial sectors, although NO and O workers have substantial differences in terms of both occupational and non-occupational exposures [28]. In some industrial sectors, the risk of thyroid cancer was higher among O workers than NO workers, the result of which was consistent with that of earlier studies [29, 30]. Various reasons could be behind this. The tendencies that O workers come from higher socioeconomic status (SES) and NO workers from lower SES were reported in some studies [31]. Workers who come from higher SES, more often O workers, can access health care services easily with private insurance, whereas NO workers face some limitation due to expenses that overwhelm their means and greater physical distances from their workplace (which tends to be of a rural location) to a hospital. We suggest that ‘over-diagnosis’ and an increased level of exposure of medical radiation owing to more frequent health check-ups are two reasons behind the high prevalence of thyroid cancer among O workers. O workers’ business naturally entails less physical activities during their work; such a lack of physical activities might affect obesity, which in turn, might affect the risk of thyroid cancer [32]. Contrary to the old concept that NO male workers are considered a higher occupational exposure group [28], occupational environment in which female NO workers work could be considered safer as of now. Swedish study observed that among workers employed in Medical and other health service, standardized incidence ratio (SIR) of male workers was high (1.82, *P* < 0.5) but that of female workers was low (0.98, *P* > 0.5) [29]. Some epidemiologic studies suggest that underrepresentation of women working in industrial workforces can be explained by tendency of female workers to work in the service sector [33]. On the contrary, 60% of female workers in Shanghai, China, engage in manufacturing industries (Wong-Ho Chow, personal communication, 2002) [34]. As the border between the role of females and males become to blur in Korean workforce, harmful occupational exposures to female workers might become more accented. The fact that the data we used did not include some of the NO female workers working in a very poor occupational environment can serve as a possible explanation as to why NO workers were not susceptible to the development of thyroid cancer when compared to O workers. Immigrant workers have become to replace Korean workers in more dangerous and precarious workplaces, especially in the field of construction, agriculture and nursing aid and at small factories [35]. Further, workers who might be considered as especially vulnerable: temporary workers, daily workers and self-employed workers were not included in the data we used for analysis.

### Industry sectors

Of all industries we studied, we noted an outstanding increase of thyroid cancer in Financial and insurance activities sector. Compared with that of general women population, the prevalence of thyroid cancer among workers employed in the sector was 3 times greater and among NO workers in Financial and insurance activities sector an AOR was marginally increased. Yue Ba et al. [36] observed an insignificantly increased AOR of thyroid cancer among financial managers (1.59, 95% CI = 0.53–4.75) but when stratification by size (< 1 cm) of papillary tumor was taken, the AOR was found to be significantly increased (4.34, 95% CI = 1.32–14.31). Yue Ba et al. insisted that “the observed association could reflect a better access to medical care because white-collar jobs are more likely to have comprehensive private health insurance and therefore detection of small tumors among them are more likely,” highlighting an important relationship between the comprehensibility of private health insurance and the rigorousness of medical screening. We hypothesize that comprehensive private health insurance that Korean female workers in a Financial and insurance activities sector have an access to might explain an outstanding increase in our study. In addition, as workers of insurance companies are required to sell a quota of insurances, they often are forced to buy insurances for themselves, leading to better insurance coverages. For this, workers can easily receive an excessive level of screening including on thyroid. While a true increase of thyroid cancer cannot be ruled out completely, stress or other unknown risk factors could affect the development of thyroid cancer. As we can observe from the result of stratified job duration and p trend analysis, the longer workers in Financial and insurance activities sector worked, the risk of thyroid cancer became higher.

Health workers have a better access to healthcare and to some, an exposure of medical radiation could be one of the risk factors [36]. However, we found no significant increase of thyroid cancer among NO workers of Human health and social work activities sector compared with O workers of the same sector (in terms of SRR). As mentioned before, in general, O workers from a majority of industrial sectors including Human health and social work activities sector have more opportunities to receive medical care and, as a result, the SPR of O workers of Human health and social work activities sector rank somewhere in the middle amongst the SPR values of other industrial sectors. On the other hand, to NO workers of Human health and social work activities sector, better access to health care as well as increased exposure to medical radiation altogether might be reasons for the increase of thyroid cancer. In recent Korean study, the risk of thyroid cancer among female workers who were exposed to high level of radiation was increased significantly [37]. In our result, the SPR of NO workers from Human health and social work activities sector ranked 3rd amongst that of NO workers of other industrial sectors. However, there could be various explanation regarding AOR that is slightly decreased, seen among NO workers in Human health and social work activities sector. First of all, the prevalence of reference population (O workers from all industrial sectors) used as a denominator in calculating AOR might be big. This would mean that for O workers in whole from all industrial sectors, gains access to medical care as easily as NO workers from Human health and social work activities do. Secondly, workers directly exposed to radiation at work could only constitute a small portion of NO workers in Human health and social work activities sector. Hence, the increased owing to the effect of radiation and an easier access to healthcare could be diluted. Finally, the degree of radiation exposure to the female workers could be less than that to the male workers, which would mean that an increase in the risk of thyroid cancer among female workers attributable to radiation exposure might not be so great after all [38].

In Agriculture, forestry, fishing, mining and quarrying sector, we found a significantly increased SPR among O workers (2.86, 95% CI = 1.05–6.22) but an insignificantly increased SPR among NO workers (1.48, 95% CI = 0.59–3.05). Several studies have been performed to find an association between the use of pesticide and thyroid cancer; a recent review revealed inconsistent, however, suggestive association [39]. In a rural/agricultural society in Korea, a lack of workers has been a serious problem; hence, O or NO workers alike all probably would have cooperated while spraying pesticide during the season. In addition, incidental exposures to O workers who are stationed near the storage of pesticide or who venture near while mixing pesticide or filling pesticide bottles is quite possible. In an agricultural/rural area, all workers, regardless of categories that their jobs entail, might have been exposed to pesticide. However, such a pertinent reports regarding forestry, fishing, mining and quarrying to substantiate our findings did not exist.

The SPR for both NO (1.42, 95% CI = 0.65–2.70) and O (2.75, 95% CI = 1.38–4.93) workers from the Manufacture of coke, briquettes, refined petroleum, chemicals and chemical products industry was significantly higher. There is a review wherein no conclusive association between solvent and thyroid cancer was ascertained [39]. However, another interesting study noted that female workers in the shoe and leather industry faced almost-doubled risk of thyroid cancer while male workers did not show such increased risk despite their more frequent exposures to solvent while working; the authors postulated that different health effects observed in response to occupational hazards between male and female might reflect their hormonal differences, which might have, in turn, resulted in female workers’ greater susceptibility to thyroid cancer [40].

Electromagnetic fields and ionizing radiation could be harmful factors of thyroid cancer to workers related with electronics industries. As Korea has remained famous in producing quality semiconductors, semiconductor workers probably occupy an appreciable portion of the workers in Manufacture of electronic components, computer; visual, sounding and communication equipment sector. One study had found a significantly increased SIR of thyroid cancer among Korean male workers in the semiconductor manufacturing industry (2.11, 95% CI = 1.49–2.89), while not among Korean female workers (0.99, 95% CI = 0.76–1.27) [41]. Our study also did not reveal any particular association between female thyroid cancer and Manufacture of electronic components, computer; visual, sounding and communication equipment sector as well. Workers in Publishing activities, motion picture, broadcasting activities, telecommunications, and information service activities were subject to a higher prevalence of thyroid cancer than general female population. Female workers in this sector might have been surrounded by electrical devices in their workplace although the industrial sector is so broad that we cannot ascertain just how much exposure to ELFMF has occurred quantitatively. While there is no consistent evidence that singles extremely low-frequency magnetic fields (ELFMF) out as a culprit of thyroid cancer [42], some studies reported the suspected risk of thyroid cancer among workers working in Video Display Terminal [43] and as electrical workers [44].

Both significantly decreased SRR and AOR were observed in Business facilities management and business support services sector, which would mean that NO workers, compared to O workers, had a lower prevalence of thyroid cancer. The major occupation in this industry, especially among female NO workers, would be that involved in cleaning. On the contrary to our result, Pukkala et al. [45] noted a significantly increased SIR of thyroid cancer among female building caretakers (1.08, 95% CI = 1.01–1.15), and Yue Ba et al. [36] also reported an increased AOR of thyroid cancer among “building and ground cleaning and maintenance workers” (OR 2.12, 95% CI = 0.99–4.54), and “building cleaners and pest control workers” (OR 2.36, 95% CI = 1.02–5.50). The logic behind such result might be that a low level of income served as a barrier to better access of health care service.

Low income serving as a barrier to an access of health care service and non-sedentary workstyle in NO workers could be possible reasons for the significantly decreased SRR in some industries such as Manufacture of textiles and apparel/Wholesale and retail/Public administration and defense; compulsory social security/Membership organizations, repair and other personal services. However, a majority of the studies on an association of thyroid cancer and occupation were based on few cases, and the results have been inconclusive [36, 39]. Previous review found an inconsistent association of thyroid cancer in textile industries, sales workers, administrative and military personnel [39].

### Weakness and strength

Misclassification error of exposure might have occurred while categorizing workers either as O or NO workers, because the company’s health manager classify workers either as O or NO workers according to their own discretion. Secondly, some categories of industrial sectors, as they were presented in the cohort data, are so broad that it was difficult to pin down on an exposure that was specific to an occupation. Thirdly, information regarding female workers’ work experience before 2007 is not included and, considering the latency of a solid cancer that is at least 10 years long [46], we could not access real occupational exposure. Furthermore, as we considered only the continuous period of work in the same industrial sector, the risk after changes in an occupation could not be considered.

When we calculated SPR, there were differences in the duration of data covered by Annual report of cancer statistics and National Female Worker Cohort. As the cohort data we used for our analysis included cases of patients who were diagnosed with thyroid cancer before 2007 and then accessed medical services between 2007 and 2014 for a routine check-up or for medication such as thyroid hormone replacement pill after total or subtotal thyroidectomy, we have thought that comparing them with the data from Annual report of cancer statistics that include newly diagnosed cancer that dates back from 1999 to 2014 would be compatible. Our study adjusted for the effect of age, smoking, alcohol ingestion, income decile, and body mass index (BMI) and a level of physical exercise. When gathering information for the exposure and confounder, large percentages (16%) of individuals were excluded due to missing data. We could not adjust for risk factors such as family history of thyroid cancer and past history of benign thyroid disease as information on them was not provided for in the cohort data we used. However, Hemminki et al. [47] noted that familial risk in thyroid cancer only accounts for 3.5% of all cases of thyroid cancer. Wong et al. [48] also pointed out that as the prevalence of prior thyroid disease was less than 2% among control women, it is quite unlikely to influence the outcome significantly. We also could not adjust for an intake level of iodine. However, as our study is on Korean population, an intake level of iodine that largely depends on the consumption of seafood, is not likely to change our results significantly. As we analyzed many industrial sectors, statistical power might be weak; therefore, the probability of chance might have increased. However, we proceeded to implement a further analysis such as a trend analysis and an analysis after stratification based on the duration of job held and the number of employees working for a company.

Another limitation is that the information on when workers were diagnosed with thyroid cancer for the first time is not included in the cohort data we used; hence we couldn’t calculate a reliable incidence rate which would have given us more information. As our data set lacked any information regarding both histologic types and sizes of the thyroid cancer, we could not carry out an analysis that might have given a better clue that would have helped us in judging whether findings of thyroid cancer is the result of an increased level of screening effect or not, as well as in helping to minimize potential misclassification errors [36].

We analyzed the data of a considerable size: one hundred and eighty thousand female workers were accounted for. Further, in addition to an internal comparison analysis among female workers in Korea, an external comparison analysis with Korea’s general female population was done as well. Taking into consideration of the differences in occupational exposures that NO and O workers face, we compared the occupational risk of thyroid cancer across 33 industrial sectors. Recall bias is absent as the information regarding workers’ occupation was registered to cohort data before the cancer was diagnosed. We also focused on the important and sensitive issue in Korea: whether an increase of thyroid cancer recently is due to ‘over-diagnosis’ or a true increase, and analyzed further on Financial and insurance activities sector that showed the highest SPR value.

## Conclusions

We found that female workers had a higher prevalence of thyroid cancer than general female population, and we observed a significant increase of thyroid cancer among NO workers compared to O workers in some industrial sectors. To elucidate further association between female workers and thyroid cancer, more studies with regards to specific occupational exposures are warranted.

## Additional files

Additional file 1: Standardized Incidence Ratio and Standardized Rate Ratio by industrial sectors and job categories (Reference: Korean female general population). (DOCX 19 kb)

Additional file 2: Dose-response trend between thyroid cancer and duration of employment in Financial and insurance activities (Reference: office workers of Financial and insurance activities regarding each respective duration of work). (DOCX 15 kb)

Additional file 3: Adjusted Odds Ratio of thyroid cancer stratified by the number of employees (Reference: office workers regarding each respective number of workers). (DOCX 18 kb)

## Abbreviations

AOR: Adjusted Odds Ratio
CI: Confidence Interval
KCD-5,6: Korean Standard Classification of Diseases
KNSO: National Statistical Office of Korea
NO: Non-office
O: Office
SPR: Standardized Prevalence Rate
SRR: Standardized Prevalence Rate Ratio
