# Supplementary material for: Thyroid cancer among female workers in Korea, 2007–2015
Source: Ann Occup Environ Med. 2018 Jul 16;30:48. doi: 10.1186/s40557-018-0259-3 (PMC6048802; doi:10.1186/s40557-018-0259-3)
Supplement: Supplementary file 1 — Standardized Incidence Ratio and Standardized Rate Ratio by industrial sectors and job categories (Reference: Korean female general population). (DOCX 19 kb) [file 40557_2018_259_MOESM1_ESM.docx]

**Additional file 1** Standardized Incidence Ratio and Standardized Rate Ratio by industrial sectors and job categories (Reference: Korean female general population)

|  | Non-/office(total) | | | |  | Non-office | | | |  | Office | | | |  | Non-office/Office | | |
| --- | --- | --- | --- | --- | --- | --- | --- | --- | --- | --- | --- | --- | --- | --- | --- | --- | --- | --- |
| Industry sectors | Cases | SIR | 95% CI | |  | Cases | SIR | 95% CI | |  | Cases | SIR | 95% CI | |  | SRR | 95% CI | |
| Agriculture, forestry, fishing, mining and quarrying | 11 | 1.70 | 0.85 | 3.05 |  | 5 | 1.17 | 0.38 | 2.73 |  | 6 | 2.74 | 1.01 | 5.97 |  | 0.43 | 0.13 | 1.40 |
| Manufacture of beverages and food products | 41 | 1.29 | 0.93 | 1.75 |  | 32 | 1.32 | 0.90 | 1.86 |  | 9 | 1.21 | 0.55 | 2.29 |  | 1.09 | 0.52 | 2.29 |
| Manufacture of textiles and apparel | 49 | 1.26 | 0.93 | 1.67 |  | 28 | 1.10 | 0.73 | 1.59 |  | 21 | 1.58 | 0.98 | 2.41 |  | 0.70 | 0.40 | 1.23 |
| Manufacture of coke, briquettes, refined petroleum, chemicals and chemical products | 19 | 1.70 | 1.02 | 2.65 |  | 8 | 1.18 | 0.51 | 2.32 |  | 11 | 2.51 | 1.25 | 4.50 |  | 0.47 | 0.19 | 1.16 |
| Manufacture of rubber and plastics products | 12 | 1.00 | 0.52 | 1.75 |  | 7 | 0.82 | 0.33 | 1.69 |  | 5 | 1.45 | 0.47 | 3.39 |  | 0.57 | 0.18 | 1.78 |
| Manufacture of basic metals | 23 | 1.16 | 0.74 | 1.75 |  | 15 | 1.17 | 0.66 | 1.94 |  | 8 | 1.14 | 0.49 | 2.26 |  | 1.03 | 0.43 | 2.42 |
| Manufacture of electronic components, computer; visual, sounding and communication equipment | 56 | 1.27 | 0.96 | 1.65 |  | 47 | 1.36 | 1.00 | 1.81 |  | 9 | 0.93 | 0.42 | 1.76 |  | 1.47 | 0.72 | 3.00 |
| Manufacture of electrical equipment | 12 | 1.15 | 0.59 | 2.00 |  | 6 | 0.85 | 0.31 | 1.86 |  | 6 | 1.74 | 0.64 | 3.80 |  | 0.49 | 0.16 | 1.52 |
| Manufacture of machinery and equipment | 116 | 1.12 | 0.93 | 1.35 |  | 94 | 1.22 | 0.98 | 1.49 |  | 22 | 0.85 | 0.53 | 1.29 |  | 1.43 | 0.90 | 2.28 |
| Manufacture of motor vehicles, trailers  and semitrailers, and transport equipment | 31 | 0.98 | 0.67 | 1.40 |  | 23 | 0.92 | 0.58 | 1.38 |  | 8 | 1.24 | 0.54 | 2.45 |  | 0.74 | 0.33 | 1.65 |
| Manufacture of wood, products of wood, cork and furniture | 11 | 1.12 | 0.56 | 2.01 |  | 6 | 0.96 | 0.35 | 2.10 |  | 5 | 1.40 | 0.46 | 3.27 |  | 0.69 | 0.21 | 2.25 |
| Other manufacturing | 51 | 1.41 | 1.05 | 1.85 |  | 36 | 1.45 | 1.02 | 2.01 |  | 15 | 1.31 | 0.73 | 2.16 |  | 1.11 | 0.61 | 2.03 |
| Construction | 63 | 1.33 | 1.02 | 1.70 |  | 16 | 1.01 | 0.58 | 1.64 |  | 47 | 1.50 | 1.10 | 1.99 |  | 0.67 | 0.38 | 1.19 |
| Wholesale and retail trade | 244 | 1.54 | 1.35 | 1.74 |  | 107 | 1.39 | 1.14 | 1.68 |  | 137 | 1.68 | 1.41 | 1.99 |  | 0.83 | 0.64 | 1.06 |
| Transportation | 40 | 1.50 | 1.07 | 2.05 |  | 20 | 1.52 | 0.93 | 2.34 |  | 20 | 1.49 | 0.91 | 2.30 |  | 1.02 | 0.55 | 1.89 |
| Accommodation and food service activities | 66 | 1.19 | 0.92 | 1.51 |  | 41 | 1.23 | 0.88 | 1.67 |  | 25 | 1.13 | 0.73 | 1.66 |  | 1.09 | 0.66 | 1.79 |
| Publishing activities, motion picture,  broadcasting activities, telecommunications,  information service activities | 55 | 1.80 | 1.36 | 2.34 |  | 16 | 1.57 | 0.90 | 2.55 |  | 39 | 1.91 | 1.36 | 2.62 |  | 0.82 | 0.46 | 1.47 |
| Financial and insurance activities | 164 | 2.67 | 2.27 | 3.11 |  | 24 | 1.93 | 1.24 | 2.87 |  | 140 | 2.85 | 2.40 | 3.37 |  | 0.68 | 0.44 | 1.04 |
| Real estate activities and rental and  leasing activities | 87 | 1.31 | 1.05 | 1.62 |  | 44 | 1.20 | 0.87 | 1.61 |  | 43 | 1.46 | 1.05 | 1.96 |  | 0.82 | 0.54 | 1.26 |
| Professional, scientific and technical activities | 66 | 1.44 | 1.11 | 1.83 |  | 18 | 1.23 | 0.73 | 1.94 |  | 48 | 1.54 | 1.13 | 2.04 |  | 0.80 | 0.46 | 1.37 |
| Business facilities management and  business support services | 102 | 1.15 | 0.94 | 1.40 |  | 61 | 1.02 | 0.78 | 1.31 |  | 41 | 1.43 | 1.02 | 1.93 |  | 0.72 | 0.48 | 1.07 |
| Public administration and defense;  compulsory social security | 406 | 1.61 | 1.45 | 1.77 |  | 57 | 1.53 | 1.16 | 1.99 |  | 349 | 1.62 | 1.45 | 1.80 |  | 0.95 | 0.72 | 1.25 |
| Education | 237 | 1.38 | 1.21 | 1.57 |  | 110 | 1.31 | 1.08 | 1.58 |  | 127 | 1.45 | 1.21 | 1.72 |  | 0.90 | 0.70 | 1.17 |
| Human health and social work activities | 221 | 1.44 | 1.25 | 1.64 |  | 148 | 1.41 | 1.19 | 1.65 |  | 73 | 1.49 | 1.17 | 1.88 |  | 0.94 | 0.71 | 1.25 |
| Arts, sports and recreation related services | 29 | 1.34 | 0.90 | 1.93 |  | 15 | 1.14 | 0.64 | 1.88 |  | 14 | 1.66 | 0.91 | 2.78 |  | 0.69 | 0.33 | 1.43 |
| Membership organizations,  repair and other personal services | 101 | 1.34 | 1.09 | 1.63 |  | 51 | 1.20 | 0.90 | 1.58 |  | 50 | 1.52 | 1.12 | 2.00 |  | 0.79 | 0.54 | 1.17 |

For age-standardized external comparison, the total of 158,569 workers, of which 74,682/83,887 workers were classified as non-office/office respectively, were enrolled between the year of 2008 and 2015 regarding their occupation and information on categorization of occupation (non-office/office) did not exist. Among them, 2,350 workers, of which 1,049/1,301 workers were classified as non-office/office respectively, were diagnosed with thyroid cancer.
