# Supplementary material for: Thyroid cancer among female workers in Korea, 2007–2015
Source: Ann Occup Environ Med. 2018 Jul 16;30:48. doi: 10.1186/s40557-018-0259-3 (PMC6048802; doi:10.1186/s40557-018-0259-3)
Supplement: Supplementary file 2 — Dose-response trend between thyroid cancer and duration of employment in Financial and insurance activities (Reference: office workers of Financial and insurance activities regarding each respective duration of work). (DOCX 15 kb) [file 40557_2018_259_MOESM2_ESM.docx]

**Additional file 2** Dose-response trend between thyroid cancer and duration of employment in Financial and insurance activities (Reference: office workers of Financial and insurance activities regarding each respective duration of work)

|  | ≤3 year | | | | | | |  | 3-8 year | | | | | | |  | >8 year | | | | | | |  |
| --- | --- | --- | --- | --- | --- | --- | --- | --- | --- | --- | --- | --- | --- | --- | --- | --- | --- | --- | --- | --- | --- | --- | --- | --- |
| Industrial sector | cases | COR* | 95% CI | | AOR**†** | 95% CI | |  | cases | COR* | 95% CI | | AOR**†** | 95% CI | |  | cases | COR* | 95% CI | | AOR**†** | 95% CI | | P for trend |
| Financial and  insurance activities | 5 | 0.51 | 0.21 | 1.25 | 0.51 | 0.20 | 1.29 |  | 5 | 0.53 | 0.22 | 1.31 | 0.56 | 0.22 | 1.40 |  | 23 | 1.11 | 0.71 | 1.73 | 1.10 | 0.70 | 1.73 | 0.076 |

***COR adjusted for age only**

**†AOR adjusted for age, smoking, alcohol, BMI, income decile, physical activity**
