# Supplementary material for: Thyroid cancer among female workers in Korea, 2007–2015
Source: Ann Occup Environ Med. 2018 Jul 16;30:48. doi: 10.1186/s40557-018-0259-3 (PMC6048802; doi:10.1186/s40557-018-0259-3)
Supplement: Supplementary file 3 — Adjusted Odds Ratio of thyroid cancer stratified by the number of employees (Reference: office workers regarding each respective number of workers). (DOCX 18 kb) [file 40557_2018_259_MOESM3_ESM.docx]

**Additional file 3** Adjusted Odds Ratio of thyroid cancer stratified by the number of employees (Reference: office workers regarding each respective number of workers)

|  | <100 workers | | | |  | ≥100 workers | | | |
| --- | --- | --- | --- | --- | --- | --- | --- | --- | --- |
| Industrial sectors | cases | AOR* | 95%CI |  |  | cases | AOR* | 95%CI |  |
| Agriculture, forestry, fishing, mining and quarrying | 3 | 1.10 | 0.35 | 3.47 |  | 4 | 1.10 | 0.41 | 2.99 |
| Manufacture of beverages and food products | 9 | 0.63 | 0.33 | 1.23 |  | 27 | 1.15 | 0.77 | 1.70 |
| Manufacture of textiles and apparel | 14 | 0.59 | 0.35 | 1.01 |  | 16 | 1.17 | 0.70 | 1.95 |
| Manufacture of rubber and plastics products | 5 | 0.63 | 0.26 | 1.52 |  | 2 | 0.44 | 0.11 | 1.79 |
| Manufacture of basic metals | 11 | 0.85 | 0.46 | 1.55 |  | 5 | 0.79 | 0.32 | 1.92 |
| Manufacture of electronic components, computer;  visual, sounding and communication equipment | 19 | 0.97 | 0.61 | 1.55 |  | 31 | 0.74 | 0.51 | 1.07 |
| Manufacture of electrical equipment | 7 | 1.13 | 0.53 | 2.40 |  | 1 | 0.22 | 0.03 | 1.57 |
| Manufacture of machinery and equipment | 46 | 1.01 | 0.74 | 1.37 |  | 57 | 0.73 | 0.55 | 0.96 |
| Manufacture of motor vehicles, trailers  and semitrailers, and transport equipment | 13 | 0.67 | 0.38 | 1.16 |  | 16 | 0.82 | 0.50 | 1.36 |
| Manufacture of wood, products of wood, cork and furniture | 5 | 0.76 | 0.31 | 1.85 |  | 2 | 0.82 | 0.20 | 3.35 |
| Other manufacturing | 12 | 0.68 | 0.38 | 1.21 |  | 27 | 0.77 | 0.52 | 1.13 |
| Construction | 12 | 0.87 | 0.49 | 1.55 |  | 7 | 0.78 | 0.36 | 1.66 |
| Wholesale and retail trade | 66 | 1.09 | 0.85 | 1.41 |  | 56 | 0.98 | 0.74 | 1.30 |
| Transportation | 2 | 0.50 | 0.12 | 2.00 |  | 19 | 0.92 | 0.58 | 1.46 |
| Accommodation and food service activities | 19 | 0.67 | 0.42 | 1.07 |  | 25 | 1.10 | 0.73 | 1.65 |
| Publishing activities, motion picture,  broadcasting activities, telecommunications,  information service activities | 2 | 0.44 | 0.11 | 1.75 |  | 17 | 1.23 | 0.76 | 2.01 |
| Financial and insurance activities | 10 | 1.83 | 0.97 | 3.46 |  | 23 | 1.20 | 0.78 | 1.83 |
| Real estate activities and rental and  leasing activities | 10 | 0.51 | 0.27 | 0.95 |  | 37 | 0.92 | 0.65 | 1.31 |
| Professional, scientific and technical activities | 11 | 1.09 | 0.60 | 1.99 |  | 9 | 0.61 | 0.31 | 1.18 |
| Business facilities management and  business support services | 18 | 0.96 | 0.59 | 1.54 |  | 48 | 0.62 | 0.46 | 0.85 |
| Public administration and defence;  compulsory social security | 13 | 0.91 | 0.52 | 1.59 |  | 46 | 0.88 | 0.65 | 1.20 |
| Education | 96 | 1.03 | 0.83 | 1.28 |  | 30 | 0.75 | 0.52 | 1.09 |
| Human health and social work activities | 105 | 0.99 | 0.80 | 1.22 |  | 69 | 0.89 | 0.69 | 1.15 |
| Arts, sports and recreation related services | 4 | 0.68 | 0.25 | 1.83 |  | 13 | 0.83 | 0.47 | 1.45 |
| Membership organizations,  repair and other personal services | 17 | 0.63 | 0.39 | 1.02 |  | 36 | 0.90 | 0.64 | 1.28 |

*AOR adjusted for age, smoking, alcohol, BMI, income decile, physical activity
